# Synthetic antibody-derived immunopeptide provides neuroprotection in glaucoma through molecular interaction with retinal protein histone H3.1

**DOI:** 10.3389/fmed.2022.993351

**Published:** 2022-10-14

**Authors:** Kristian Nzogang Fomo, Carsten Schmelter, Joshua Atta, Vanessa M. Beutgen, Rebecca Schwarz, Natarajan Perumal, Gokul Govind, Thomas Speck, Norbert Pfeiffer, Franz H. Grus

**Affiliations:** ^1^Department of Experimental and Translational Ophthalmology, University Medical Center, Johannes Gutenberg University, Mainz, Germany; ^2^Institute of Physics, Johannes Gutenberg University, Mainz, Germany

**Keywords:** glaucoma, retina, autoimmunity, neuroprotection, synthetic CDR peptides

## Abstract

Glaucoma is a group of optic neuropathies characterized by the progressive degeneration of retinal ganglion cells (RGCs) as well as their axons leading to irreversible loss of sight. Medical management of the intraocular pressure (IOP) still represents the gold standard in glaucoma therapy, which only manages a single risk factor and does not directly address the neurodegenerative component of this eye disease. Recently, our group showed that antibody-derived immunopeptides (encoding complementarity-determining regions, CDRs) provide attractive glaucoma medication candidates and directly interfere its pathogenic mechanisms by different modes of action. In accordance with these findings, the present study showed the synthetic complementary-determining region 2 (CDR2) peptide (INSDGSSTSYADSVK) significantly increased RGC viability *in vitro* in a concentration-dependent manner (*p* < 0.05 using a CDR2 concentration of 50 μg/mL). Employing state-of the-art immunoprecipitation experiments, we confirmed that synthetic CDR2 exhibited a high affinity toward the retinal target protein histone H3.1 (HIST1H3A) (*p* < 0.001 and log2-fold change > 3). Furthermore, molecular dynamics (MD) simulations along with virtual docking analyses predicted potential CDR2-specific binding regions of HIST1H3A, which might represent essential post-translational modification (PTM) sites for epigenetic regulations. Quantitative mass spectrometry (MS) analysis of retinas demonstrated 39 proteins significantly affected by CDR2 treatment (*p* < 0.05). An up-regulation of proteins involved in the energy production (e.g., ATP5F1B and MT-CO2) as well as the regulatory ubiquitin proteasome system (e.g., PSMC5) was induced by the synthetic CDR2 peptide. On the other hand, CDR2 reduced metabolic key enzymes (e.g., DDAH1 and MAOB) as well as ER stress-related proteins (e.g., SEC22B and VCP) and these data were partially confirmed by microarray technology. Our outcome measurements indicate that specific protein-peptide interactions influence the regulatory epigenetic function of HIST1H3A promoting the neuroprotective mechanism on RGCs *in vitro*. In addition to IOP management, such synthetic peptides as CDR2 might serve as a synergistic immunotherapy for glaucoma in the future.

## Introduction

Glaucoma is a slowly progressing ocular disorder mainly characterized by the loss of retinal ganglion cells (RGCs) and their axons resulting in irreversible blindness of the patients (1). RGCs are the primary output neurons of the retina which process and relay the visual information from the retina to the brain. Due to that reason, RGCs are reliable anatomical markers for glaucoma model systems to evaluate the effectiveness of potential glaucoma medications (2). However, current therapeutic standard therapies still target the management of the elevated intraocular pressure (IOP), which is a main characteristic in the pathogenesis of glaucoma (1). Nevertheless, these therapeutic procedures just address a clinical sign of glaucoma and do not directly intervene the pathogenic neurodegenerative mechanism of this complex eye disease. Even though, brimonidine an IOP-lowering drug compound approved for glaucoma treatment has recently shown direct retinal neuroprotection in animal paradigm of RGC damage (3). Furthermore, newest findings demonstrate the great potential of caffeine for RGC neuroprotection with its anti-inflammatory and anti-oxidative properties (4) illustrating new perspectives for the medical management of retinal diseases in the future. In accordance with that, our research group focuses the application of antibody-based immunotherapeutics as promising starting point for the treatment of glaucoma, which specifically target pathogenic key molecules in the retina such as HMGB1 (5) or α-synuclein (6) and provide ocular neuroprotection by different modes of action. Since the pharmaceutical trend nowadays is toward the development of small drug compounds with enhanced bioactivity (7), the downsizing of large biomolecules and the restriction to only biologically active binding sites has become of great interest for clinical manufacturing.

Hitherto, the application of therapeutic peptides mimicking the active binding sites of antibodies has been mainly used in cancer research, particularly to treat melanoma (8, 9). Thereby, these peptides encoded the complementary-determining regions (CDRs) of the antibodies, which determine the specificity and affinity to the respective antigen. Interestingly, the antitumorigenic functions of the synthetic CDR peptides are independent of the antigen specificity of the native antibodies and are supposed to present a natural, unlimited source of potentially bioactive drug compounds (10). One of these immunopeptides, termed as C36L1, triggered antitumorigenic effects by interfering the actin cytoskeleton organization as well as the cell cycle regulation in melanoma cells (10) and by eliciting specific immunoregulatory functions (11). Therapeutic peptide AC-1001 H3, in contrast, induced cytotoxic effects in melanoma cells by triggering the formation of reactive oxygen species (ROS) as well as the occurrence of autophagy-related mechanism (12) and mirrors the versatile therapeutic modes of actions of these immunopeptides.

In this context, our research group focuses on the medical application of antibody-derived immunopeptides in neurodegenerative diseases with a special focus on glaucoma. Recently, our group provided strong evidence that the antibody-derived immunopeptide ASGYTFTNYGLSWVR (encoding the CDR1 of IGHV1-18*02; identified in Schmelter et al., (13) provide neuroprotection on RGCs in a retina organ culture model *in vitro*. In follow-up study, our data indicated neuroprotection by ASGYTFTNYGLSWVR was likely due to the physical interaction with serine protease HTRA2 (14). Furthermore, the synthetic CDR peptide significantly inhibited the proteolytic activity of HTRA2 *in vitro* and seems to affect important HTRA2-mediated signaling pathways in the retina such as aggrephagy or cellular metabolism (15). As conclusion, it can be assumed that the peptide-induced HTRA2 protease inhibition seems to be a beneficial mechanism for RGC neuroprotection. These data indicate the great potential of synthetic immunopeptides for the medical management of glaucoma as well as other neurodegenerative-associated diseases.

In accordance with this line of evidence, here we investigated the therapeutic effects of synthetic immunopeptide INSDGSSTSYADSVK [encoding the complementary-determining region 2 (CDR2) of IGHV3-74*01] in the retina organ culture model *in vitro* to evaluate its potential neuronal tissue-preservative functions. The premise for this investigation is strong in that CDR2 peptide was initially identified as potential biomarker candidate in the serum of glaucoma patients and expression was significantly reduced in the systemic IgG molecule structure in comparison to healthy controls (13). The main goal of the present study was to identify direct retinal interaction partners of synthetic CDR2 using affinity capture mass spectrometry (AC-MS) and to characterize its neuroprotective molecular mechanism in glaucoma by quantitative proteomics. The results of this study will provide deeper insights into the pathophysiology of glaucoma and may form the basis for a synergistic peptide immunotherapy in the future. As main result, the present study clearly showed that the synthetic immunopeptide INSDGSSTSYADSVK elicited significant RGC neuroprotection *in vitro* and potentially caused these beneficial effects by physical interaction with retinal target protein histone H3.1 (HIST1H3A).

## Materials and methods

### Synthetic complementary-determining region 2 peptides

The CDR2 peptide (INSDGSSTSYADSVK) is derived from polyclonal, sera-derived antibody molecules, which were originally identified as potential biomarker candidates in POAG patients (13). The synthetic peptides for the experiments were purchased from the company Synpeptide Co., Ltd., (Shanghai, China) with a purity of >90%. The peptides were synthesized as followed: INSDGSSTSYADSVK (without modification) and Biotin-[Acp]-INSDGSSTSYADSVK. The synthetic peptide with the N-terminal Biotin-[Acp] modification was used for the identification of CDR-specific interaction partners (see the section “Identification of complementary-determining region 2-specific interaction partner”) and the unmodified peptide was used for the *in vitro* retina organ culture model (see the section “Molecular dynamics simulations and docking analysis”).

### Identification of complementary-determining region 2-specific interaction partner

For the identification of CDR-specific interaction partners in the retina, we used retinal protein homogenate from the house swine (*Sus scrofa domestica* Linnaeus, 1,758) for the co-immunoprecipitation experiments. The eye bulbs (*n* = 30) were provided by local slaughterhouses (Landmetzgerei Harth, Stadecken Elsheim, Germany) and the protein homogenate was prepared as described elsewhere in detail (14). The application of animal by-products of the house swine for research purposes was approved by the Kreisverwaltung Mainz–Bingen in Germany (Identification Code: DE 07 315 0006 21, approved on the 13th January 2014). In brief, the retinal tissues were extracted from the eye bulbs and subjected to further protein homogenization protocol as mentioned above. The resulting retinal protein homogenate was pooled and stored in 5 mg aliquots at 20^°^C before further processing. For the identification of CDR2-specific interaction partners, the synthetic peptide Biotin-[Acp]-INSDGSSTSYADSVK was immobilized on Pierce™ Streptavidin Magnetic Beads (Thermo Fisher Scientific, Rockford, IL, USA) according to the manufacturer’s instructions. Therefore, 50 μL of the streptavidin magnetic beads were washed twice with 200 μL of phosphate-buffered saline (PBS) using a magnetic stand followed by an incubation with 80 μg of the biotin-labeled peptide for 1 h at RT. Control beads (CTRL) were labeled with 0.5 mg/mL biotin (Thermo Fisher Scientific, Rockford, IL, USA) for 1 h at RT to distinguish non-specific from CDR2-specific protein binders. After peptide immobilization, the magnetic bead fractions (*n* = 3 per group) were washed twice with PBS followed by incubation and gentle mixing with 5 mg homogenized porcine retina at 4^°^C overnight. Next day, the magnetic beads were washed three times with 300 μL of PBS and the remaining attached proteins were eluted with 100 μL of Pierce™ IgG Elution buffer pH 2.0 (Thermo Fisher Scientific, Rockford, IL, USA). The eluate fractions were neutralized with 10 μL of 1 M Tris HCl pH 8.5 and subsequently evaporated in the speed vacuum concentrator (SpeedVac; Eppendorf, Darmstadt, Germany) for 30 min at 30^°^C until dryness. Afterward, the samples were stored at -20^°^C prior to further in-solution trypsin digestion.

### Molecular dynamics simulations and docking analysis

The initial configurations of the amino acid sequence were modeled using PyMOL molecular graphic system version 1.8 (Schrödinger, LLC, Portland, OR, USA) and all molecular dynamics (MD) simulations were performed using GROMACS 2021. The peptide was equilibrated for 2 ns in both NVT and NPT ensembles using SPC/E water as the explicit solvent. Then, each MD simulation was carried out for 200 ns. To improve sampling, five independent 200 ns simulations initialized with random velocities were performed for the peptide. Snapshots were rendered using visual molecular dynamics (VMD) version 1.9.4 (Theoretical and Computational Biophysics Group, Beckman Institute, Urbana, IL, USA) and all figures were plotted using python. Autodock Vina version 1.1.2 (Center for Computational Structural Biology, San Diego, CA, USA) was used to predict potential CDR2-specific binding regions of HIST1H3A (PDB protein structure: 2CV5; chain: C). The peptide secondary structure used for the docking binding energy calculation was obtained from the MD simulations.

### Retina organ culture model and immunohistology

The retina organ culture model was prepared from eye bulbs of the house swine (*Sus scrofa domestica* Linnaeus, 1,758) as described elsewhere in detail (14). In brief, 5 × 5 mm retina–retinal pigment epithelium (RPE) complexes were prepared under sterile conditions and cultured for 24 h at 37^°^C and 5% CO2. Following the 24 h *in vitro*, we have previously observed significantly and reproducible degeneration of RGCs *in vitro* caused by axotomy due to optic nerve cut (ONC) (14, 16). The retina–RPE complexes were either untreated as controls or treated with 25 and 50 μg/mL of the synthetic CDR2 peptide (INSDGSSTSYADSVK), respectively (*n* = 5 for each group). After cultivation, the respective control and peptide-treated retinal sections were carefully removed from the RPE, washed twice with PBS and fixed in 4% paraformaldehyde (PFA) for 30 min at RT. Afterward, the retinal explants were washed twice with PBS and subsequently blocked for 2 h with 200 μL of blocking buffer (0.3% Triton-X-100 and 10% of fetal calf serum (FCS) in PBS). The FCS was purchased from Merck Millipore (Darmstadt, Germany). Then, the retinal explants were washed two more times with PBS and incubated with goat anti-Brn3a (Santa Cruz Biotechnology, Dallas, TX, USA) in a ratio of 1:250 in 10% FCS PBS at 4^°^C overnight. Next day, the retinal explants were washed twice with PBS and incubated with Alexa Flour 568 donkey anti-goat (H + L; Thermo Fisher Scientific, Rockford, IL, USA) in a ratio of 1:400 in 10% FCS PBS for 2 h in the dark. After three more washing steps, the retinal tissues were stained with 4’,6-Diamidin-2-phenylindol (DAPI; Thermo Fisher Scientific, Rockford, IL, USA) in a ratio of 1:2,500 in PBS for 5 min at RT. Subsequently, the retinal tissues were washed and flat-mounted with vector shield mounting medium (Vector Laboratories, Burlingame, CA, USA) on Superfrost Plus™ slides (Thermo Fisher Scientific, Rockford, IL, USA). For the microscopic analyses, an Eclipse TS100 fluorescence microscope (Nikon Instruments Inc., Melville, USA) coupled with a DS-Fi1-U2 digital camera (Nikon Instruments Inc., Melville, USA) was used. Nine high-resolution fluorescent images (20-fold magnification) were taken from each retinal explant at different positions resulting in 45 fluorescent images for each group (TRITC and DAPI channel). The high-resolution fluorescent images were randomized and analyzed by experienced laboratory workers with software tool ImageJ (17). Brightness and contrast of the images were adjusted to facilitate the manual counting of the Brn3a^+^ cells and the number of RGCs (Brn3a^+^ cells) was extrapolated to RGC/mm^2^ for each group.

### In-solution trypsin digestion

For the identification of CDR-specific protein interaction partners, the eluate fractions of both bead groups (see the section “Identification of complementary-determining region 2-specific interaction partner”) were subjected to further in-solution trypsin digestion and peptide purification as previously described in Schmelter et al., (14). For the quantitative proteomic analysis of the untreated and CDR-treated retinal explants (see the section “Molecular dynamics simulations and docking analysis”), the sample preparation protocol was slightly modified. At first, the respective untreated and CDR-treated retinal explants were cultured for 24 h at 37^°^C and 5% CO2 (*n* = 3 for each group) and were subsequently snap-frozen in liquid-nitrogen. Then, the frozen retinal tissues ± treatment were subjected to further homogenization and protein extraction protocol as ascribed in (14). In brief, 400 μL of T-PER™ buffer (Thermo Fisher Scientific, Rockford, IL, USA) and 1.4/2.8 mm ceramic balls were added to each sample and treated with the Precellys^®^ 24 homogenizer (VWR, International GmbH, Darmstadt, Germany). Then, the protein-containing supernatant was exchanged into 200 μL of PBS using an Amicon 3 kDa centrifugal filter device (Millipore, Billerica, MA, USA). A protein measurement of each sample was performed using the Pierce BCA Protein Assay Kit and the Multiscan Ascent photometer (Thermo Fisher Scientific, Rockford, IL, USA) and 10 μg of each sample were evaporated in the SpeedVac at 30^°^C until dryness. The proteins were resolved in 30 μL of 10 mM ammonium bicarbonate (ABC) and 3 μL of 20 mM dithiothreitol (DTT) in 10 mM ABC were added followed by incubation for 30 min at 56^°^C. Next, 3 μL of 40 mM iodoacetamide (IAA) in 10 mM ABC were added and incubated for 30 min at RT in the dark. The reduced and alkylated proteins were digested with 1 μg of trypsin (Promega, Madison, WI, USA) in 10 mM ABC 10% acetonitrile (ACN) at 37^°^C overnight and next day quenched with 10 μL of 0.1% formic acid (FA). All samples were evaporated in the SpeedVac at 30^°^C until dryness and peptide purification was performed using SOLAμ™ HRP SPE spin plates (Thermo Fisher Scientific, Rockford, IL, USA) as previously described (14).

### LC-MS/mass spectrometry analysis

All Liquid chromatography-mass spectrometry (LC-MS/MS) measurements were performed with the Hybrid Linear Ion Trap-Orbitrap MS system (LTQ Orbitrap XL; Thermo Fisher Scientific, Rockford, IL, USA). For the proteomic analysis of the CDR-specific interaction partners (see the section “Identification of complementary-determining region 2-specific interaction partner”), the LTQ Orbitrap XL MS was coupled to an capillary-HPLC system system as previously described (13, 14). Information about HPLC settings and MS parameters for the analysis are listed in detail in Schmelter et al., (14). For the quantitative proteomic analysis of the retina organ culture model ± treatment (see the section “Molecular dynamics simulations and docking analysis”), the LTQ Orbitrap XL MS was online coupled to an EASY-nLC 1200 system (Thermo Fisher Scientific, Rockford, IL, USA) combined with a PepMap C18 column system (75 mm × 500 mm; Thermo Fisher Scientific, Rockford, IL, USA). For this analysis, solvent A consisted of 0.1% FA in water and solvent B consisted of 0.1% FA in 80% ACN. Peptides were eluted within 320 min using a flow rate of 0.3 μL/min with the following gradient: 5–30% B (0–290 min), 30–100% B (290–300 min), and 100% B (300–320 min). The MS system operated with a resolution of 30,000 in the positive ion mode and the target automatic gain control (AGC) was set to 1 × 10^6^ ions. The lock mass for internal calibration was set to 445,120,025 m/z (polydimethylcyclosiloxane). The dynamic exclusion (DE) parameters were enabled with the following settings: repeat count = 1, repeat duration = 30 s, exclusion list size = 100, exclusion duration = 300 s, and exclusion mass width of ±20 ppm. Furthermore, collision-induced decay (CID) fragmentation was performed for the five most intense precursor ions using a normalized collision energy of 35%. The MS proteomics data have been deposited to the ProteomeXchange Consortium *via* the PRIDE (18) partner repository with the dataset identifier PXD034046.

### Protein identification and quantification

The LC-MS raw data were analyzed with the bioinformatics software MaxQuant version 1.6.17.0 (Max Planck Institute for Biochemistry, Martinsried, Germany). Protein identification was performed with reviewed SwissProt databases using the taxonomies *Homo sapiens* (date: 13/04/2021, number of sequences: 20,408) and *Sus scrofa* (date: 13/04/2021, number of sequences: 1,439). Due to limited access to proper public proteomic databases of the house swine (*Sus scrofa*) (19), we included the species-related proteomic database of *Homo sapiens* to increase protein identifications. The following parameters were used for the database search: peptide ion mass tolerance of ±30 ppm, ion fragment mass tolerance of 0.5 Da, tryptic cleavage, maximum of two missed cleavage sites, carbamidomethylation as fixed modification, acetylation (N-terminal protein) and oxidation as variable modifications. The MaxQuant-specific feature “match between run” was enabled and all proteins were filtered with a false discovery rate (FDR) < 1%.

### Microarray analysis

Microarray production was performed in-house, using a non-contact array printer (SciFLEXARRAYER S3; Scienion, Berlin, Germany). Antibodies for proteins of interest (see Table 1) were spotted on nitrocellulose-covered microarray slides (AVID Oncyte, 16 Pad NC slides; Grace Bio-Labs, Bend, OR, USA) in triplicates. Retina lysates from CDR2-treated and untreated control samples (*n* = 3 per group) were labeled with a fluorescent dye (DyLight 650 NHS Ester; Thermo Fisher Scientific, Rockford, IL, USA). Therefore, retinal lysates with a total protein amount of 100 μg per sample were prepared in labeling buffer (0.05 M sodium borate buffer, pH 8.5). Subsequently, the samples were incubated then with 1 μL of dye solution for 1 h at RT in the dark. In addition, a negative control containing only labeling buffer as well as a positive control comprising a pooled sample were also included in this experiment. The labeling reaction was stopped by addition of 10 μL of quenching solution (Tris-HCl, pH 8.8) and incubation for 30 min at RT in the dark. The unbound dye was removed by using Zeba desalt plates (Zeba Spin Desalting Plates, 7k MWCO; Thermo Fisher Scientific, Rockford, IL, USA) according to the manufacturer’s instructions. For hybridization, the prepared array slide was mounted into a 16 well incubation chamber (ProPlate Multiwell chambers; Grace Bio-Labs, Bend, OR, USA). The following incubation steps were carried out at 4^°^C on an orbital shaker. First, the slide was incubated with blocking buffer (Super G; Grace Bio-Labs, Bend, OR, USA) for 1 h to improve the signal-to-noise ratio. Afterward, the blocking buffer was discarded, and the slide was washed four times with PBST (PBS with 0.5% Tween-20). Afterward, the slide was incubated with 100 μL of labeled sample per subarray for 2 h at RT. One subarray was incubated only with PBS as additional negative control. After this step, the residual sample was removed, and the slide was washed two times with PBST and two times with ultra-pure water. Finally, the slide was dried for 2 min in the SpeedVac at 30^°^C. Subsequently, the slide was imaged using a CCD-camera based array reader (SensoSpot; Sensovation, Radolfzell, Germany). Then, the slide was scanned at 25 and 100 ms exposure time in the red channel. Images were recorded as 16-bit TIF files. Spot intensities were quantified using Imagene (Imagene 5.5; BioDiscovery Inc., Los Angeles, CA, USA). Spots that did not meet quality control criteria were flagged and removed from further analysis. Before statistical evaluation, microarray data were pre-processed. Therefore, local background intensity was removed from spot intensity to calculate net signal intensities and signals from triplicate spots were averaged.

**TABLE 1:** List of antibodies used for the microarray analysis.

Antibody target	Host species	Manufacturer
ALDH3A2	Rabbit	Proteintech GMBH, Manchester, UK
BCL2L10	Rabbit	Cell Signaling Europe, Frankfurt, Germany
BAD	Rabbit	Cell Signaling Europe, Frankfurt, Germany
BAX	Rabbit	Abcam, Boston, MA, USA
Beclin-1	Rabbit	Cell Signaling Europe, Frankfurt, Germany
DDAH1	Rabbit	MyBioSource, Inc., San Diego, CA, USA
HAX1	Rabbit	Biorbyt Ltd., Cambridge, UK
XIAP	Rabbit	Cell Signaling Europe, Frankfurt, Germany

### Statistical analysis and bioinformatics

The statistical analysis of the proteomics data was performed with the software program Perseus v. 1.6.15 (Max Planck Institute for Biochemistry, Martinsried, Germany). At first, the data were filtered for contaminants, reversed hits as well as “only identified by site.” Then, the intensities of the detected proteins were log2-transformed. True protein hits were required to be detected in at least three biological replicates of one study group (CTRL or CDR-treated group) and were considered for further statistical analysis. Missing protein intensity values were imputed from the normal distribution of the data (width: 0.3, down shift: 1.8). For the identification of CDR-specific interaction partners (see the section “Molecular dynamics simulations and docking analysis”), only missing values of the control bead group (CTRL) were imputed in accordance with the previous publication (14). Afterward, two-sided *t*-test statistics for pairwise comparison was performed and proteins with *p*-values < 0.05 were considered as statistically significant. For the illustration of the heat map, the protein abundances were standardized by *Z*-score and the hierarchical clustering was based on Euclidean distance using the Perseus software package. In addition, further statistical analyses and graphical presentation of the data were performed with Statistica version 13 (Statsoft; Tulsa, OK, USA). Furthermore, the functional annotation of the proteins and the signaling pathway analyses were performed with open-source biological database STRING version 11.5 (Search Tool for the Retrieval of Interacting Genes/Proteins) and Ingenuity Pathway Analysis software v. 1-04 (IPA, Ingenuity QIAGEN; Redwood City, CA, USA) as previously described (14, 20). Statistical analyses of the microarray data and further graphical presentation of the proteomic data were performed with Statistica version 13 (Stat soft; Tulsa, OK, USA). Two-sided *t*-test statistics for pairwise comparison of the quantitative microarray data was used to determine significantly changed protein markers (*p* < 0.05).

## Results

### Identification of complementary-determining region 2-specific interaction partners

**FIGURE 1:**
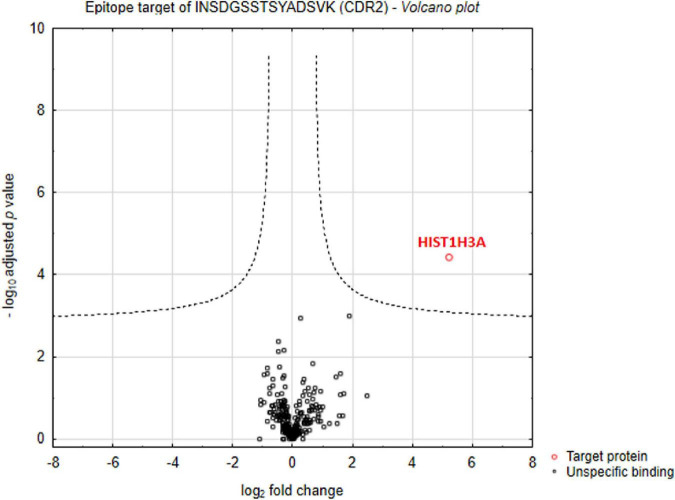
Identification of complementary-determining region 2 (CDR2)-specific interaction partners in porcine retina. Volcano plot shows log2-fold change as function of -log10-adjusted *p*-values for samples from the CDR2-coupled bead group versus samples from the biotin-coupled control bead group (*n* = 3 per group; *p* < 0.001 and log2-fold change > 3). The protein histone H3.1 (HIST1H3A) was identified as major protein interaction partner of the synthetic CDR2 peptide (INSDGSSTSYADSVK).

First of all, we were interested in direct interaction partners of synthetic CDR2 in retinal tissues of the house swine (*Sus scrofa domestica*) and we have performed an MS-based immunoprecipitation experiment as previously described (14). For that reason, the biotin-labeled CDR2 peptide was coupled to magnetic beads and incubated for 24 h with homogenized porcine retina. In addition, a biotin-labeled control bead group was also included in this analysis to distinguish unspecific from CDR-specific protein binders. After incubation, both bead fractions ± CDR2 were intensively washed, and the eluate fractions were screened for potential protein interaction partners by LC-MS analysis. The statistical analysis of the proteomic data revealed that the protein histone H3.1 (HIST1H3A) represents a major protein interaction partner of the synthetic CDR2 peptide (INSDGSSTSYADSVK) (*p* < 0.001 and log2-fold change > 3; see Figure 1 and Supplementary material 1).

### Molecular dynamics simulations and molecular docking analysis of the complementary-determining region 2 peptide

**FIGURE 2:**
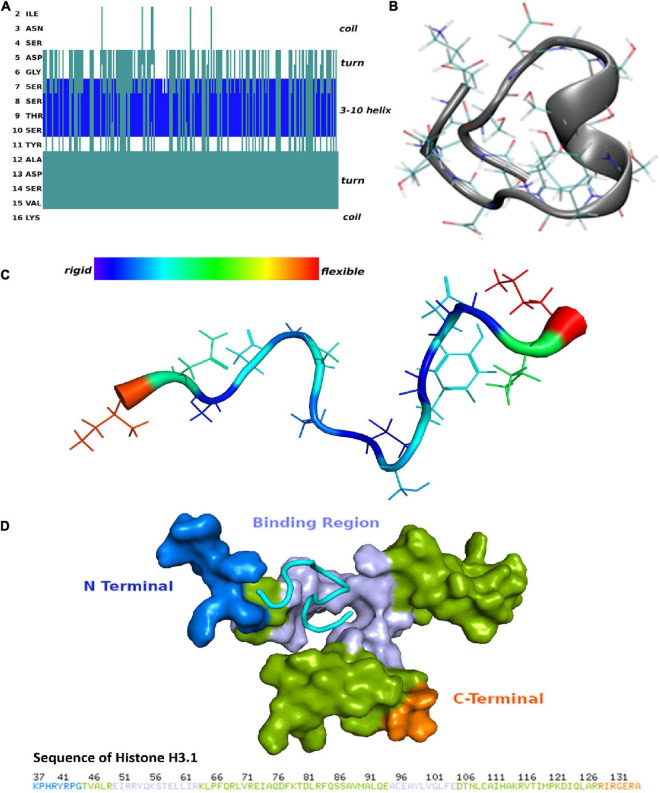
Secondary structure of the complementary-determining region 2 (CDR2) peptide (INSDGSSTSYADSVK) and prediction of potential binding sites to retinal target protein histone H3.1 (HIST1H3A). **(A)** Structuring of the CDR2 peptide revealed by the molecular dynamics (MD) simulations. **(B)** Secondary structure of the CDR2 peptide showing a rigid 3–10 helix portion. The central helical region is formed by the amino acids ^5^DGSSTSY^11^. **(C)** Flexibility of the CDR2 peptide though peptide backbone fluctuations during MD simulations. The central helical regions including the turns show a rigid structural behavior, whereas the coil parts of the peptide indicate a high flexibility. **(D)** Potential binding site of the CDR2 peptide to target protein HIST1H3A from molecular docking calculations. The peptide interacts with the N-terminal part [from E (50) to R (63)] as well as with the C-terminal region [A (95) to E (105)] of protein HIST1H3A (PDB protein structure: 2CV5; chain: C).

To determine the secondary structure characteristics of the synthetic CDR2 peptide (INSDGSSTSYADSVK) and to evaluate the importance of these specific intramolecular interactions for the binding to the target protein histone H3.1 (HIST1H3A), we performed MD simulations and molecular docking analyses *in silico* (see Figures 2A–D and Supplementary Figure 1). The most probable peptide structure from the equilibrium MD simulations comprised a 3-10 helix portion, which is connected to a turn on either side and is followed then by random coil tails (see Figure 2A). Thereby, the central helical region of the CDR2 peptide is comprised of the amino acid sequence D (5)-GSSTS-Y(11) and its secondary structure is shown in Figure 2B. The helical and turn regions of the peptide show a rigid structural behavior, whereas the coil parts are much more flexible (see Figure 2C). Considering this important structural information, we performed further molecular docking analyses to the experimentally determined target protein HIST1H3A (PDB protein structure: 2CV5; chain: C). It should be noted that all RCSB protein structures contain no coordination data for the N-terminal part of this protein, because it is unstructured and highly modified. However, the *in silico* molecular docking analysis (see Figure 2D) predicted a significant binding site of CDR2 to the N-terminal part [from E (50) to R (63)] as well as to the C-terminal region [from A (95) to E (105)] of the PDB protein structure HIST1H3A (2CV5, chain: C). Particularly, the rigid helical structure of the peptide including the stabilized turn regions seem to be an important structural feature for this specific protein-peptide interaction. In conclusion, these results are in accordance with the experimental data and support a physical interaction between the synthetic CDR2 peptide and the retinal target protein HIST1H3A.

### Complementary-determining region 2 peptides induce a neuroprotective effect on pig retinal ganglion cells

**FIGURE 3:**
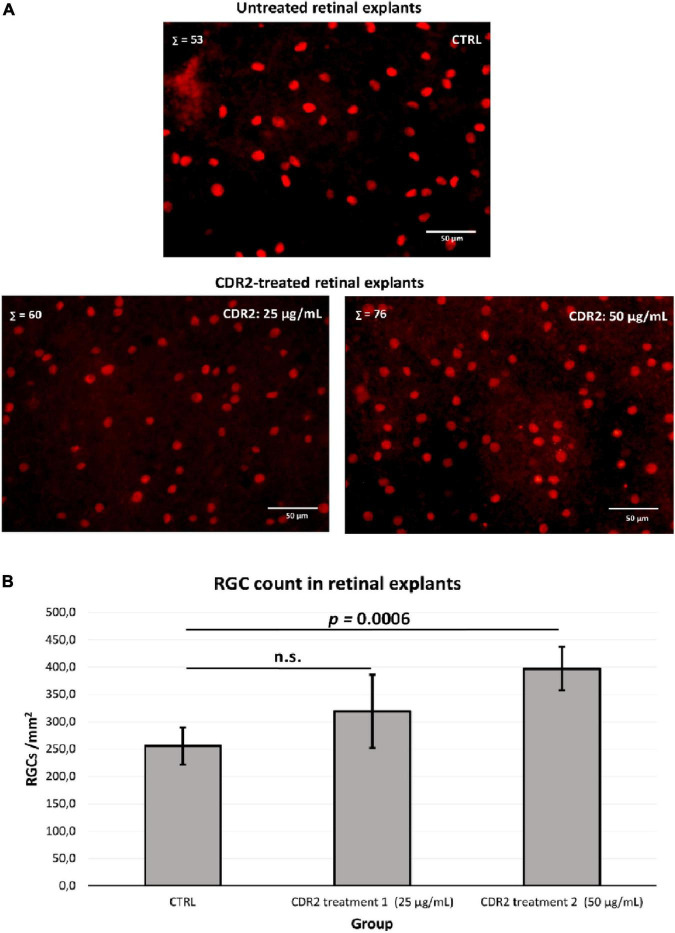
Evaluation of the neuroprotective effects of the synthetic complementary-determining region 2 (CDR2) peptide (INSDGSSTSYADSVK) on the viability of retinal ganglion cells (RGCs) in the retina organ culture model *in vitro*. **(A)** The retinal explants (*n* = 5 per group) were treated with either 25 or 50 μg/mL CDR2 peptide or remained untreated as control group. The Brn3a^+^ immunostaining was performed for the microscopic detection of RGCs. Σ represents the number of Brn3a^+^ RGCs in each picture. **(B)** The quantitative analysis of Brn3a^+^ cells (RGCs) showed no significant difference between the retinal explants treated with 25 μg/mL of synthetic CDR2 compared to control group (*p* > 0.05). Ascending the concentration of synthetic CDR2 to 50 μg/mL induced a significant higher survival rate of Brn3a^+^ RGCs in the CDR2-treated retinal explants compared to the respective control explants (*p* = 0.0006).

To evaluate the potential neuroprotective effects of the synthetic CDR2 peptide (INSDGSSTSYADSVK), we used the *in vitro* retina organ culture model of the domestic pig (*Sus scrofa domesticus*). The stress factor in this model system was induced by mechanical separation of the optic nerve from the retina, which led to a significant and reproducible RGC loss after 24 h of incubation under physiological conditions (14). Brn3a is a reliable molecular marker for the microscopic detection of RGCs and is routinely used to assess the neuroprotective properties of potential glaucoma medications (2). Therefore, retinal explants were treated with either 25 or 50 μg/mL of the synthetic CDR2 peptide or remained untreated as control group (CTRL, *n* = 5 per group). High-resolution images of the Brn3a immunostaining ± CDR2 treatments are shown in Figure 3A. The quantitative analysis (see Figure 3B) of Brn3a^+^ cells revealed no significant difference in retinal explants treated with 25 μg/mL of synthetic CDR2 (319.4 ± 67 RGCs/mm^2^) compared to untreated CTRL (289.3 ± 61.8 RGCs/mm^2^, *p* > 0.05). On the other hand, ascending the concentration of synthetic CDR2 to 50 μg/mL resulted in a significant higher viability of Brn3a^+^ cells in the CDR2-treated explants (382.9 ± 44.9 RGCs/mm^2^) compared to the respective untreated controls (232.4 ± 18.8 RGCs/mm^2^, *p* < 0.05, see Figure 3B).

### LC-MS analysis of retinal explants

**FIGURE 4:**
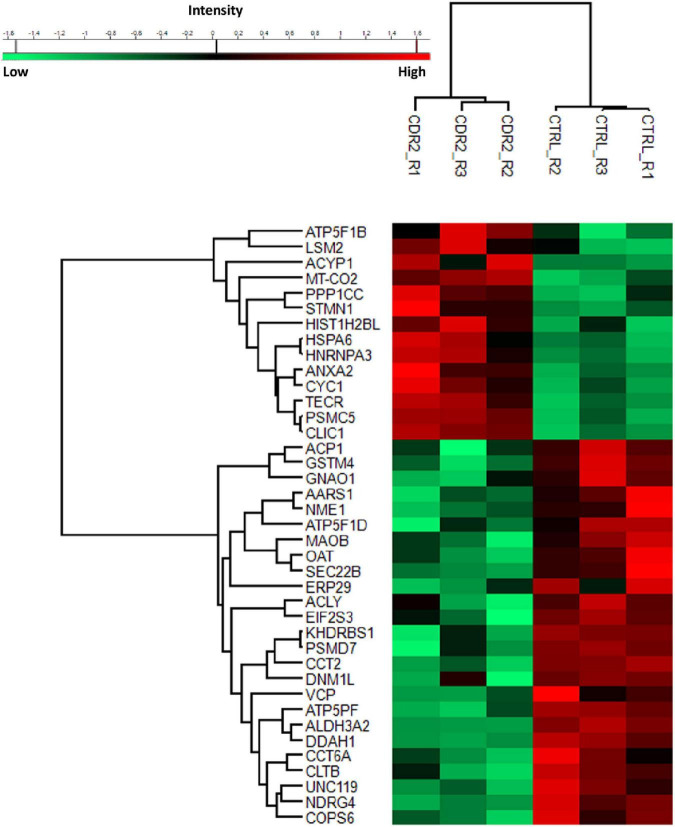
Differential expression levels of significantly changed protein markers (*p* < 0.05) in complementary-determining region 2 (CDR2)-treated retinal explants compared to untreated controls (CTRL). The highly expressed proteins are shown in red color and the low abundant proteins are labeled in green color between both experimental groups (CDR2 and CTRL group).

**FIGURE 5:**
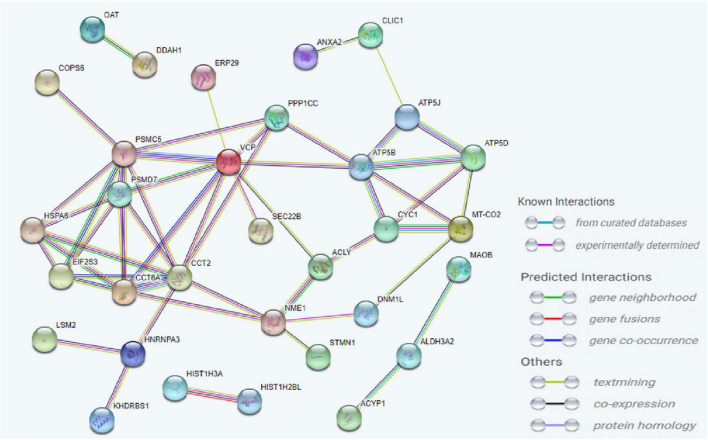
Analysis of the interaction network of the most significantly altered proteins between complementary-determining region 2 (CDR2)-treated and untreated retinal explants (*p* < 0.05). The STRING functional protein association network was used for the protein-protein interaction analysis. The chromatin-associated protein HIST1H2BL was identified as a reliable interaction partner of CDR2-specific target protein HIST1H3A.

To investigate the molecular mechanism of the peptide-induced neuroprotective effects on RGCs *in vitro*, we performed a quantitative LC-MS-based proteomic analysis of the retinal explants ± CDR2 treatment (*n* = 3 per group). Because the neuroprotective effects on RGCs were significantly enhanced with ascending amounts of the peptide, we chose a concentration of 50 μg/mL of synthetic CDR2 for this experiment. Overall, 942 proteins were identified in either the CDR2-treated or the untreated control explants (FDR < 1%). Of these proteins, 39 were significantly affected by CDR2 treatment, with 14 up- and 25 down-regulated proteins in the CDR2-treated group compared to untreated CTRL (*p* < 0.05, see Figure 4 and Supplementary material 2). Interestingly, many proteins, which were found in high abundance in the CDR2-treated explants compared to CTRL, were associated with the energy production (e.g., ATP synthase subunit beta, ATP5F1B and cytochrome c oxidase subunit 2, and MT-CO2) and protein folding such as 26S proteasome regulatory subunit 8 (PSMC5) (see Table 2). On the contrary, chaperone-like proteins (e.g., T-complex protein 1 subunit beta and CCT2), metabolic enzymes (e.g., N (G), N (G)-dimethylarginine dimethylaminohydrolase 1, and DDAH1) and ER-stress related proteins (e.g., vesicle-trafficking protein SEC22b, SEC22B) were down-regulated in the CDR2-treated explants compared to untreated CTRL. Box plots of selected marker proteins are shown in Supplementary Figure 2. Furthermore, the IPA signaling pathway analysis revealed that Huntington’s disease (*p* = 1.88 ⋅10^–8^), mitochondrial dysfunction (*p* = 3.81 ⋅10^–7^), oxidative phosphorylation (*p* = 1.07 ⋅10^–6^), BAG2 signaling pathway (*p* = 1.20 ⋅10^–5^), and the sirtuin signaling pathway (*p* = 1.25 ⋅10^–4^) are the most affected canonical pathways in the CDR2-treated retinal explants (see Table 3). However, we could not identify the CDR2-specific target protein histone H3.1 (HIST1H3A) in this proteomic MS analysis. Nevertheless, another chromatin-associated protein histone H2B type 1-L (HIST1H2BL), which also represents a core component of the nucleosome, was identified as significant high abundant protein marker in the CDR2-treated explants compared to untreated controls (*p* = 0.02, see Figure 4 and Supplementary material 2). Moreover, a functional annotation and pathway analysis *via* biological STRING database revealed an experimentally determined interaction between protein marker HIST1H2BL and CDR2-specfic target HIST1H3A (see Figure 5). In addition, the other significantly changed protein markers also showed a complex protein interaction network as shown in Figure 5.

**TABLE 2:** List of molecular and cellular functions [derived from Ingenuity Pathway Analysis (IPA) analysis] associated with differentially expressed proteins in both experimental groups.

Molecular and cellular functions	*p*-value range	Molecules
Energy production	2.99 × 10^–2^–8.76 × 10^–9^	11
Nucleic acid metabolism	3.65 × 10^–2^–8.76 × 10^–9^	14
Small molecule biochemistry	4.45 × 10^–2^–8.76 × 10^–9^	20
Post-translational modification	3.64 × 10^–2^–8.92 × 10^–8^	14
Protein folding	3.64 × 10^–2^–8.92 × 10^–8^	6

**TABLE 3:** List of top affected canonical pathways in both experimental groups.

Canonical pathway	*p*-value	Molecules
Huntington’s disease signaling	1.88 × 10^–8^	ATP5F1B, ATP5F1D, ATP5PF, CLTB, DNM1L, HSPA6, PSMC5, and PSMD7
Mitochondrial dysfunction	3.81 × 10^–7^	ATP5F1B, ATP5F1D, ATP5PF, CYC1, MAOB, and MT-CO2
Oxidative phosphorylation	1.07 × 10^–6^	ATP5F1B, ATP5F1D, ATP5PF, CYC1, and MT-CO2
BAG2 signaling pathway	1.20 × 10^–5^	ANXA2, HSPA6, PSMC5, and PSMD7
Sirtuin signaling pathway	1.25 × 10^–4^	ACLY, ATP5F1B, ATP5F1D, ATP5PF, and CYC1

### Validation of selected protein markers using microarray technology

**FIGURE 6:**
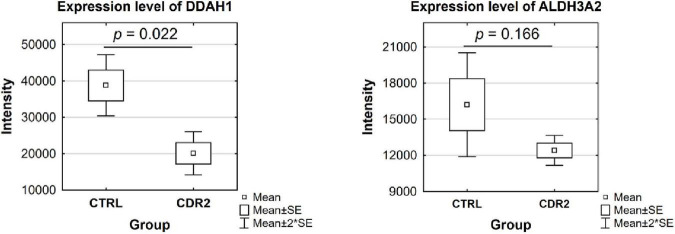
Validation of marker proteins DDAH1 and ALDH3A2 by microarray technology. The box plots highlight the expression level of the proteins validated by microarray. The selected proteins show similar trends to the results obtained by MS analysis. DDAH1 was identified with significantly lower abundance in the CDR2-treated group compared to untreated CTRL (*p* < 0.05). The ALDH3A2 protein showed a tendency to be lesser expressed in the CDR2-treated explants compared to CTRL but was not supported by statistical significance (*p* ¿ 0.05).

Based on candidate proteins revealed by MS analysis, we quantified some of these proteins using microarray technology in both experimental groups (CTRL and CDR2 group). In addition, we also quantitatively analyzed both groups for the expression of apoptosis-related (BAD, BAX, BCL2L10, and XIAP) and autophagy-associated (HAX1 and Beclin-1) marker proteins, since both signaling cascades are supposed to be highly involved in the molecular pathogenesis of glaucoma (21). Statistical analysis of the microarray data revealed that the DDAH1 protein was significantly reduced in the CDR2-treated explants compared to untreated controls (*p* = 0.022, see Figure 6) similar to the results of the MS analysis. Also, CDR2 tended to decrease ALDH3A2 expression in the CDR2-treated explants compared to untreated CTRL, but was not supported by statistical significance (*p* = 0.166, see Figure 6). The statistical analysis of the apoptosis-related and autophagy-associated proteins also revealed slightly lesser abundances in CDR2-treated group compared to CTRL for all markers, but were also not supported by statistical significance (*p* > 0.05, see Supplementary Figure 3).

## Discussion

The establishment of new, more effective and less toxic therapies/medications for chronic diseases, particularly glaucoma, remains to be a major challenge for modern medicine and pharmacology. The development of therapeutic substances based on endogenous molecules of the human body represents a considerable progress in the creation of new drug candidates and the implementation of personalized medicine. Thus, active regulators such as synthetic peptides from the CDR of antibodies represent a major strategy in this new type of research due to their small size, low immunogenicity, and good penetration properties into cells as well as the nucleus (22). In addition, they are easy and cheap to manufacture and provide a wide range of structural modification possibilities to improve their physicochemical characteristics (e.g., to increase the plasma half-lives). Moreover, several studies already reported that synthetic CDR peptides possess a wide variety of regulatory activities such as immunomodulatory, antimicrobial, antiviral as well as antitumor functions and represent excellent therapeutic agents for the treatment of cancer, particularly melanoma (8, 9).

The present study was designed to characterize retinal interaction partners of the synthetic CDR2 peptide (INSDGSSTSYADSVK), which was previously identified as low abundant peptide marker in the serum of POAG patients (13). Furthermore, it was scheduled to evaluate if the synthetic CDR2 peptide exhibits whether neuroprotective or even neurotoxic effects on RGCs *in vitro*. Applying MS-based immunoprecipitation experiments, we proved that the synthetic CDR2 peptide exhibits a high affinity for the protein histone H3.1 (HIST1H3A), which was highly enriched from the retinal proteome of the domestic pig (see Figure 1 and Supplementary material 1). Thereby, the target protein HIST1H3A represents a subunit of the nucleosome complex, which is formed by a histone octamer (including H3 dimers) and a stretch of DNA. In general, histones are positively charged proteins interacting with DNA in the nucleus promoting the condensation of DNA into chromatin. Accordingly, they play an important role in the gene regulation (23). During the gene transcription process, the structure of chromatin can be altered by various post-translational modifications (PTMs) of the histones resulting in increased relaxation or condensation of the DNA. Thus, these epigenetic processes allow transcription factors and RNA polymerases to access the genes (24). The virtual docking analysis along with the MD simulations revealed that the CDR2 peptide most probably binds to the globular domain [comprising the sequence parts from E (50) to R (63) and A (95) to E (105)] of target protein HIST1H3A in a secondary structure-dependent manner (see Figure 2). Interestingly, this CDR2-specific binding site comprises the important PTM residue K56 for acetylation of HIST1H3A, which was already associated with important regulatory functions such as genomic stability, chromatin assembly, DNA replication, cell cycle progression, and DNA repair in mammals (25, 26). The authors provided evidence that cell lines harboring unmodified K56 of HIST1H3A possess drastic morphological changes as well as cell death, whereas acetylation at the same position reversed these effects (25, 26). These findings highlight the important regulatory function of this PTM site for the genomic stability and the DNA damage response. Possibly, the binding of synthetic CDR2 to this PTM site elicited specific epigenetic regulations of HIST1H3A, which are beneficial for RGC neuroprotection during glaucomatous conditions. In addition, other PTM sites (e.g., K64) close to the CDR2 binding region, might be also affected in this context (27). In the literature it is very well documented that peptides can serve as important epigenetic modulators (e.g., by blocking DNA methylation or histone deacetylation) (28) and are in accordance with our binding hypothesis. Furthermore, several PTM sites of histone H3 were already associated with various pathological processes in neurodegenerative diseases such as Alzheimer’s and Parkinson’s disease (29, 30). With respect to the eye, Zhao et al. (31) postulated that PTMs of histones are able to modulate photoreceptor differentiation and degeneration. However, the exact epigenetic function of HIST1H3A is still unknown in the pathophysiology of glaucoma and could open new perspectives for the medical management of this eye disease in the future.

Finally, we were able to show that the synthetic CDR2 peptide (INSDGSSTSYADSVK) exerted significant neuroprotective effects on RGCs *in vitro* using the retina organ culture model of the domestic pig. Thereby, the mean number of RGCs per mm^2^ was significantly higher (*p* < 0.05) in explants treated with synthetic CDR2 in comparison to untreated CTRL in a concentration-dependent manner (see Figure 3). This *in vitro* model is routinely used by our group for glaucoma-related studies (14, 16), as it shows topographic similarity to the human retina and the stress-induced RGCs loss is suitable to evaluate the efficiency of potential glaucoma therapeutics (32). Furthermore, to gain deeper insight into the molecular mechanisms, which elicit these beneficial neuroprotective effects, we performed an MS-based proteomic analysis of the CDR2-treated explants compared to untreated CTRL group (see Figure 4 and Supplementary material 2). The statistical analysis of the data revealed a considerable increase in proteins related to the mitochondrial respiratory chain and energy production like ATP synthase subunit beta (ATP5F1B) and cytochrome c oxidase subunit 2 (MT-CO2) in the CDR2-treated retinal explants. Protein marker ATP5F1B is a subunit of the ATP synthase, which catalyzes the formation of the energy-carrying molecule, adenosine triphosphate (ATP), from adenosine diphosphate (ADP) and inorganic phosphate (Pi) (33). The maintenance of neuronal activity, which consists in generating sequences of action potentials for the transmission of nervous information, requires a relatively high quantity of energy (34); thus, a dysfunction of the ATP production system could dramatically interfere the neurological function and lead to increased neuronal cell death. In accordance, Richter et al. (35) reported that the maintenance of sufficient ATP levels might prevent cell apoptosis. Similarly, MT-CO2 is a subunit of the multimeric enzyme cytochrome c oxidase of the mitochondrial respiratory chain. It plays a fundamental role in the ATP formation process by catalyzing the transfer of electrons from cytochrome c to molecular oxygen and by facilitating the translocation of protons from the mitochondrial matrix to the intermembrane space. This proton translocation creates an electrochemical gradient that is used by ATP synthase to produce ATP (33). Thus, it has been shown that an energetic imbalance in retinal cells is associated with glaucoma, and may be one of the possible causes of RGC death (36). Inversely, dysfunction of the cytochrome c oxidase enzyme is associated with increased mitochondrial ROS production and cellular toxicity (37). In addition, Ko et al. (38) showed an increase in ROS levels in the retina and led to RGCs loss in an experimental glaucoma model. The scavenging or reduction of ROS resulted in an improvement of RGCs viability *in vitro* (39). In accordance with these findings, mitochondrial dysfunction and oxidative phosphorylation were identified among as the most affected CDR2-induced signaling pathways in the retina (see Table 3). Interestingly, the acetylation state of H3K56 was already associated with the epigenetic regulation of the cellular energy metabolism by sirtuins (histone deacetylases) (40), which was also identified as significant CDR2-induced signaling cascade in the present study (see Table 3).

However, other up-regulated marker proteins in the CDR2-treated explants were 26S proteasome regulatory subunit 8 (PSMC5) and Annexin A2 (ANXA2). PSMC5 is a protein of the ubiquitin-proteasome system, which is a key player in various fundamental cellular processes such as regulation of cell cycle progression, division, development and differentiation, apoptosis, cell trafficking, and the modulation of immune and inflammatory responses (41). But its main function is the targeting and degradation of damaged, oxidized or misfolded proteins in the cell (41), and is also reported to be an important regulatory mechanism in glaucoma (42). Also, ANXA2 is a protein of the annexins family that exerts a protective function on cells by regulating ROS levels and inhibiting inflammation through the release of anti-inflammatory cytokines (43). ANXA2 has been shown to regulate the immune response by inhibiting activation and brain infiltration of peripheral leukocytes after traumatic brain injury (44). Moreover, ANXA2 is able to inhibit apoptosis by suppressing the expression of pro-apoptotic genes and by triggering the synthesis of anti-apoptotic proteins (45). It was also shown that overexpression of ANXA2 can protect cells from nutrient deprivation through activation of autophagy (46). Nevertheless, it should be noted that there were no significant differences in the expression levels of apoptosis-related (e.g., BAX and BAD) and autophagy-associated proteins (e.g., Beclin-1) between both experimental groups (CTRL and CDR2, see Supplementary Figure 3).

On the other hand, we also identified protein markers which were significantly lower expressed in the CDR2-treated explants compared to untreated CTRL such as N (G), N (G)-dimethylarginine dimethylaminohydrolase (DDAH1) (*p* < 0.05, see Figure 4). Thereby, the significant lower abundance of DDAH1 was further validated by microarray technology in the peptide-treated retinal explants (see Figure 6). The DDAH1 protein represents a brain-expressed enzyme that modulates the nitric oxide pathway and is responsible for the metabolism of methylated arginines like asymmetric dimethylarginine (ADMA) and monomethylarginine (L-NMMA) (47, 48). Interestingly, several studies showed that ADMA and L-NMMA exert an inhibitory effect on the enzymes of the nitric oxide synthase family (49). The exogenous administration of ADMA and L-NMMA was reported to increase the vascular resistance and the arterial pressure in rats (50) and to promote the formation of ROS in human endothelial cells *in vitro* (51, 52). Moreover, the overexpression of ADMA in plasma or in serum was linked to cardiovascular diseases (53), atherosclerosis (54), cerebrovascular disease (55), and glaucoma (56). Conversely, low levels of ADMA were reported in diseases such as amyotrophic lateral sclerosis (57), multiple sclerosis (58), and Alzheimer’s disease (59). Since the ADMA levels were significantly increased in the serum as well as plasma of glaucoma patients (56), the CDR2-induced down-regulation of DDAH1 might indicate a lesser cellular stress response of retinal cells, particularly RGCs, *in vitro*. In addition, increased levels of L-NMMA induced by administration of an nitric oxide synthase inhibitor have been associated with an increased mortality of patients with an septic shock in a clinical trial (60).

Another low abundant marker protein in the CDR2-treated explants was amine oxidase B (MAOB), which represents an enzyme of the outer mitochondrial membrane. There it catalyzes the oxidative deamination of various primary, secondary, and tertiary amines resulting in the generation of hydrogen peroxide (H_2_O_2_) as minor product. This increased oxidative stress leads to mitochondrial dysfunction and neuronal cell death (61, 62). Increased MAOB activity might be involved in pathophysiological processes of certain neurodegenerative diseases such as Alzheimer’s and Parkinson’s diseases (63, 64). It has been shown that MAOB inhibition induced a protective effect on neurons by reducing oxidative stress and preventing mitochondrial dysfunction (65). Accordingly, its CDR2-induced down-regulation might play a key role in the RGC neuroprotection mechanism. Another low abundant marker in the CDR2-treated explants was vesicle-trafficking protein SEC22b (SEC22B), which is localized in the ER and the Golgi apparatus involved in membrane fusion events (66). It also regulates the anterograde and retrograde transport of vesicles between the ER and Golgi apparatus (67). Similarly, the CDR2-induced less abundant protein marker transitional endoplasmic reticulum ATPase (VCP) is involved in dislodging and chaperoning ubiquitinated proteins from the ER to the cytosol, where they undergo proteasomal degradation (68). Wójcik et al. (69, 70) reported that VCP seems to be key regulator during ER stress by promoting the unfolded protein response (UPR) resulting in increased degradation rates of misfolded ER proteins. In addition, a further study demonstrated that acute, chronic optic nerve injury induces ER stress in RGCs by uncontrolled accumulation of unfolded or misfolded protein structures. Thus, therapeutic molecules interfering ER stress signaling could exert remarkable protective effects on RGCs. Consequently, the targeted down-regulation of ER stress-related proteins (e.g., VCP and SEC22B, see Figure 5) might explain the neuroprotective effects of synthetic CDR2 on RGCs *in vitro.*

## Conclusion

In conclusion, the present study clearly showed that the synthetic CDR2 peptide (INSDGSSTSYADSVK) induced neuroprotective effects on RGCs *in vitro* and represents an attractive strategy for translational intervention in the future. Particularly, due to the molecular interaction of the synthetic CDR2 peptide with the target protein HIST1H3A and the binding to specific regulatory PTM sites, might explain these neuroprotective effects. It might be hypothesized that the protein-peptide interaction leads to specific epigenetic modification profiles of HIST1H3A regulating beneficial gene transcription events in the CDR2-treated retinal explants. This could explain the changed protein expression profiles involved in energy production, the ubiquitin-proteasome system regulation as well as the immune response modulation, which are important components of the CDR2-induced RGC neuroprotection mechanism *in vitro*. Nevertheless, the findings of the present study should be further validated in IOP-dependent glaucoma animal models in the future, since the retina organ culture model only mirrors the main characteristics of the complex pathophysiology of glaucoma such as progressive RGC loss. Furthermore, how exactly the CDR2 peptide influences the PTM profiles of HIST1H3A and if this changes epigenetics as well as nucleosome remodeling has also to be addressed in future studies.

## Data availability statement

The original contributions presented in the study are included in the article/Supplementary material, further inquiries can be directed to the corresponding author.

## Author contributions

KF participated in the development of the study design and the experimental workflow, performed the statistical analyses, and wrote the manuscript. CS developed the study design and the experimental workflow, performed the statistical analyses, and assisted in writing the manuscript. JA performed the experimental workflow and assisted in the data and statistical analyses. VB established and performed the microarray analysis, reviewed the manuscript, and provided important intellectual input. RS assisted in the microarray analysis and reviewed the manuscript. NPe assisted in the mass spectrometric analysis, reviewed the manuscript, and provided important intellectual input. GG developed and performed the molecular dynamics simulations as well as docking analysis and reviewed the manuscript. TS and NPf reviewed the manuscript and provided important intellectual knowledge. FG participated in study coordination and management and revised the manuscript critically. All authors have read and agreed to the published version of the manuscript.

## Supplementary material

Supplementary Figure 1 A two-dimensional number distribution of radius of gyration (RG) and root mean square displacement (RMSD) of the synthetic complementary-determining region 2 (CDR2) peptide is given in the figure with the cluster of peptide structure as insets. Among the five representative clusters, it can be observed that the helical peptide cluster is the most populated structure (RMSD: 1.35 nm and RG 0.530 nm). This helical structure of the peptide is encountered most of the times in our simulations compared to the other structures given in the figure. Peptide clustering is made using GROMACS clustering tool with a RMSD cut of 0.3 nm.

Supplementary Figure 2 Box plots showing the expression level of some significant altered proteins identified between complementary-determining region 2 (CDR2)-treated and untreated (control) retinal explants (*n* = 3 per group) by mass spectrometry (MS) (*p* < 0.05).

Supplementary Figure 3 Box plots highlighting expression level of selected apoptotic and autophagic markers identified by microarray. Additional experiments were performed to study the involvement of apoptosis and autophagy in the neuroprotective effect induced by complementary-determining region 2 (CDR2) peptides. Statistical analysis of the expression of the different markers revealed no significant difference between both experimental groups (*n* = 3 per group; *p* > 0.05).

Supplementary Material 1 Complementary-determining region 2 (CDR2)-specific interaction partners identified by mass spectrometry (MS) and statistical analysis of the MS data.

Supplementary Material 2 Mass spectrometry (MS) identification of proteins in complementary-determining region 2 (CDR2)-treated and untreated explants as well as statistical analysis of the proteins significantly altered between both groups.
