# Supplementary figures and images for: Synthetic antibody-derived immunopeptide provides neuroprotection in glaucoma through molecular interaction with retinal protein histone H3.1
Source: Front Med (Lausanne). 2022 Oct 14;9:993351. doi: 10.3389/fmed.2022.993351 (PMC9613933; doi:10.3389/fmed.2022.993351)

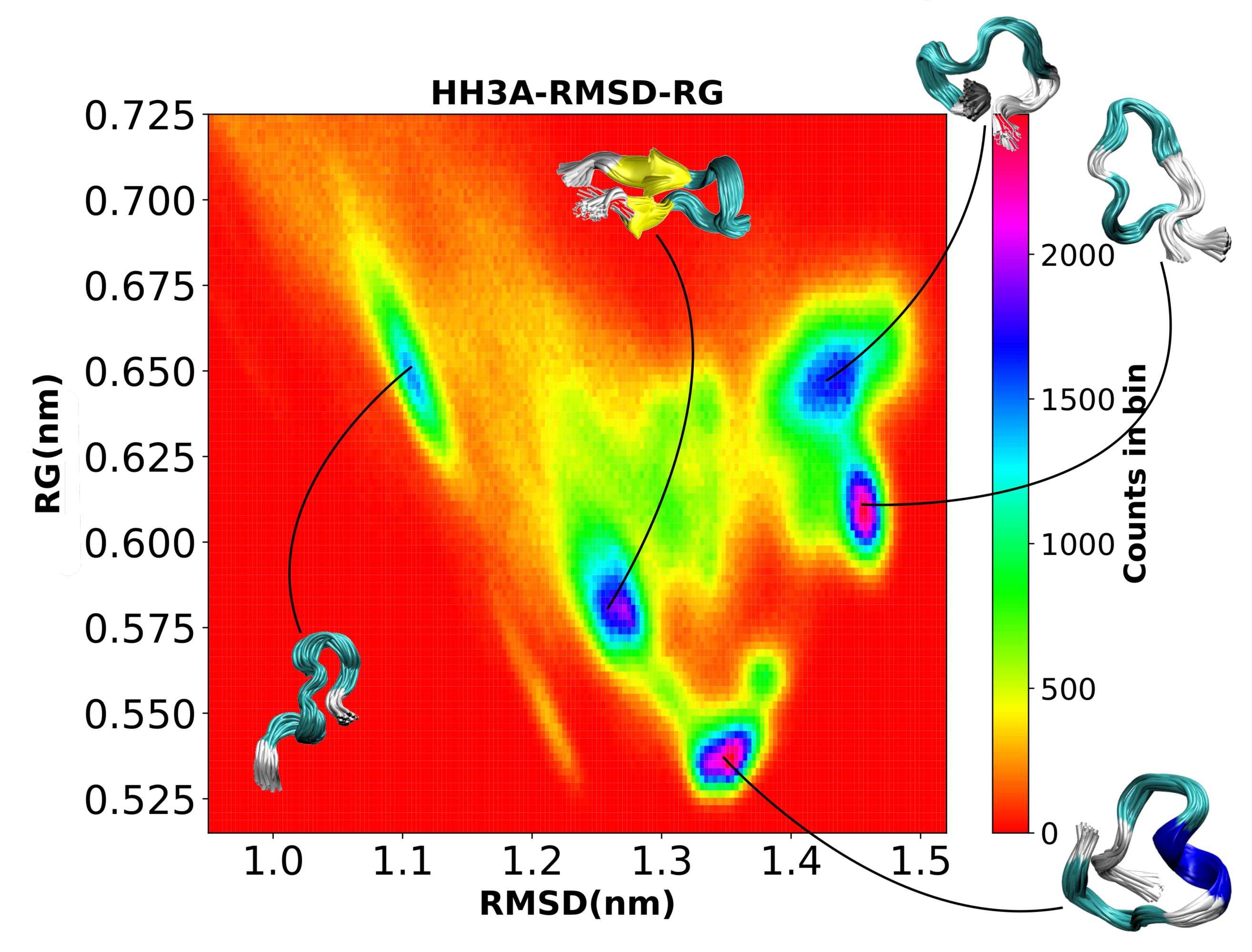

Supplement: Supplementary Figure 1 — A two-dimensional number distribution of radius of gyration (RG) and root mean square displacement (RMSD) of the synthetic complementary-determining region 2 (CDR2) peptide is given in the figure with the cluster of peptide structure as insets. Among the five representative clusters, it can be observed that the helical peptide cluster is the most populated structure (RMSD: 1.35 nm and RG 0.530 nm). This helical structure of the peptide is encountered most of the times in our simulations compared to the other structures given in the figure. Peptide clustering is made using GROMACS clustering tool with a RMSD cut of 0.3 nm. [file Image_1.TIF]

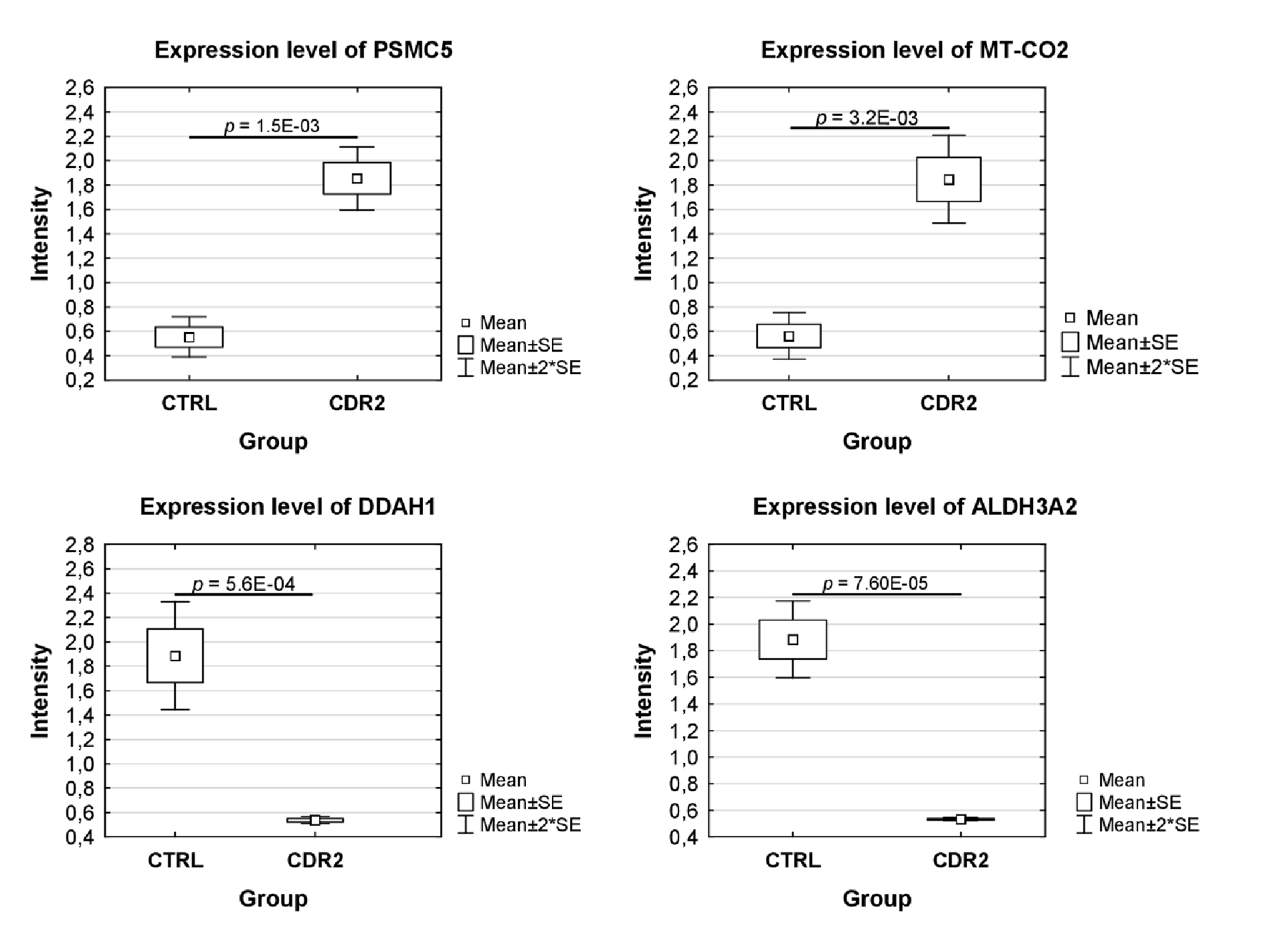

Supplement: Supplementary Figure 2 — Box plots showing the expression level of some significant altered proteins identified between complementary-determining region 2 (CDR2)-treated and untreated (control) retinal explants (n = 3 per group) by mass spectrometry (MS) (p < 0.05). [file Image_2.TIF]

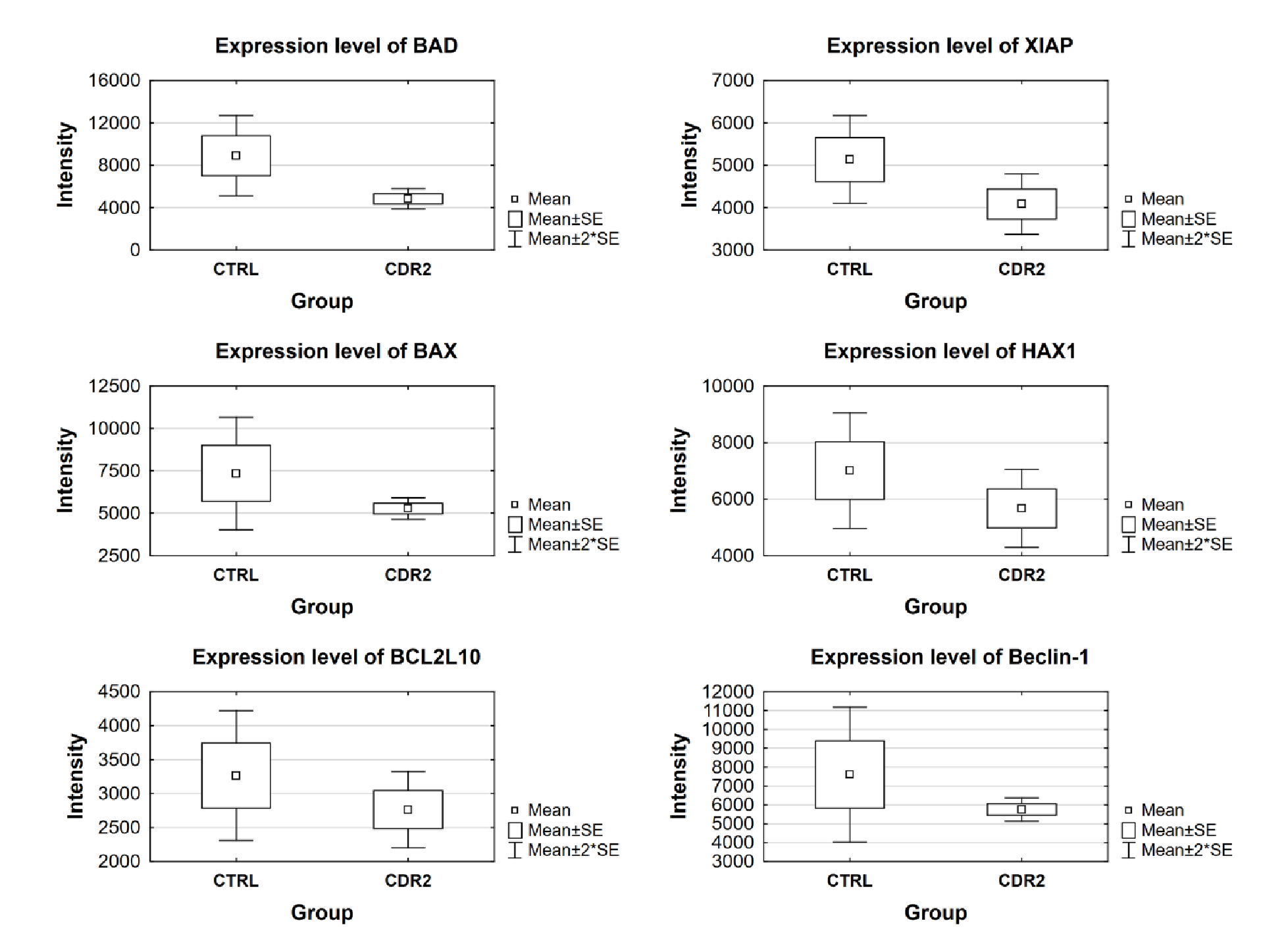

Supplement: Supplementary Figure 3 — Box plots highlighting expression level of selected apoptotic and autophagic markers identified by microarray. Additional experiments were performed to study the involvement of apoptosis and autophagy in the neuroprotective effect induced by complementary-determining region 2 (CDR2) peptides. Statistical analysis of the expression of the different markers revealed no significant difference between both experimental groups (n = 3 per group; p > 0.05). [file Image_3.TIF]
